# Microhabitat Selection by Marine Mesoconsumers in a Thermally Heterogeneous Habitat: Behavioral Thermoregulation or Avoiding Predation Risk?

**DOI:** 10.1371/journal.pone.0061907

**Published:** 2013-04-12

**Authors:** Jeremy J. Vaudo, Michael R. Heithaus

**Affiliations:** Department of Biological Sciences, Florida International University, North Miami, Florida, United States of America; University of Sydney, Australia

## Abstract

Habitat selection decisions by consumers has the potential to shape ecosystems. Understanding the factors that influence habitat selection is therefore critical to understanding ecosystem function. This is especially true of mesoconsumers because they provide the link between upper and lower tropic levels. We examined the factors influencing microhabitat selection of marine mesoconsumers – juvenile giant shovelnose rays (*Glaucostegus typus*), reticulate whiprays (*Himantura uarnak*), and pink whiprays (*H. fai*) – in a coastal ecosystem with intact predator and prey populations and marked spatial and temporal thermal heterogeneity. Using a combination of belt transects and data on water temperature, tidal height, prey abundance, predator abundance and ray behavior, we found that giant shovelnose rays and reticulate whiprays were most often found resting in nearshore microhabitats, especially at low tidal heights during the warm season. Microhabitat selection did not match predictions derived from distributions of prey. Although at a course scale, ray distributions appeared to match predictions of behavioral thermoregulation theory, fine-scale examination revealed a mismatch. The selection of the shallow nearshore microhabitat at low tidal heights during periods of high predator abundance (warm season) suggests that this microhabitat may serve as a refuge, although it may come with metabolic costs due to higher temperatures. The results of this study highlight the importance of predators in the habitat selection decisions of mesoconsumers and that within thermal gradients, factors, such as predation risk, must be considered in addition to behavioral thermoregulation to explain habitat selection decisions. Furthermore, increasing water temperatures predicted by climate change may result in complex trade-offs that might have important implications for ecosystem dynamics.

## Introduction

Habitat selection, the hierarchical process by which an animal decides which habitats to use at different scales of the environment [1], is one of the myriad decisions mobile organisms must make on a daily basis, and is critical in determining ecological dynamics at multiple scales [2]. Habitat selection is dependent on the interplay of a variety of factors. Resource quality and abundance often vary with habitat and can influence energy intake rates, thereby driving habitat selection by consumers, which, all else being equal, often attempt to maximize energy intake rates by selecting habitats with abundant, high-quality resources [3]. Predation risk and competition, on the other hand, may cause consumers to abandon otherwise productive habitats [4]–[7]. Reproductive behaviors can also influence habitat choice because habitats may vary in their benefits for spawning or the rearing of young [8]–[10].

Physiology – and how it varies across abiotic conditions – will also play a large role in habitat selection because physiological constraints may restrict access to some habitats or modify relative costs and benefits of habitats such that habitat selection does not match expectations derived simply from food supplies. This phenomenon may be especially important in aquatic systems. Salinity tolerances play a large role in the distribution and habitat selection of organisms in estuarine systems [11], [12], as does the ability to tolerate hypoxic conditions [13], [14]. One of the most important environmental factors that interacts with an organism's physiology, however, is environmental temperature because it is a key determinant of physiological performance within poikilothermic organisms [15].

Although non-optimal temperatures can negatively impact organisms, many poikilotherms can maintain a preferred temperature in a heterogeneous thermal environment by altering their behaviors, such as habitat choice [16], [17]. Because the optimal temperatures are likely to vary among metabolic processes, organisms may also gain energetically by shuttling between habitats of different temperatures [18]–[20]. Thus, habitat choice within a thermal gradient may be temperature- and behavior-dependent.

Understanding how various biotic and abiotic factors influence habitat selection by organisms, and their relative importance, is crucial to understanding systems because habitat use patterns structure the spatial and temporal pattern of interspecific interactions [21]–[23]. Such a functional understanding of habitat selection is particularly important at this time in order to predict the consequences of large-scale and ongoing changes in abiotic conditions (e.g., climate change, frequency and intensity of hypoxic events [24], [25]) as well as biotic ones (e.g., overfishing and habitat modifications [26]). Of particular interest is the dynamics of habitat selection by mesoconsumers (consumers of intermediate trophic position), which provide the link between upper and lower trophic levels and can play a major role in ecosystem structure and function through their habitat use patterns [27], [28]. For example, changes in habitat selection and the resulting foraging patterns of elk (*Cervus elaphus*) since the reintroduction of wolf (*Canis lupus*) are hypothesized to be responsible for the recovery of riparian communities, including beaver and bird populations, and ecosystem function in Yellowstone National Park, USA [29] and alteration of microhabitat selection in a grassland food web induced by elevated temperature have been shown to transform a two predator system into an intraguild predation system resulting in the loss of one of the predator species with indirect effects on plant species composition [30].

Marine mesoconsumers, however, have received less attention than their terrestrial counterparts, although they may also play important roles in community structure [27], [31]–[33]. And for some mesoconsumer species, such as winter skate (*Leucoraja ocellata*), recent local population increases have been attributed to distributional shifts possibly in response to temperature and changing trophic dynamics (i.e., large-scale habitat shifts [34]). Examining the interplay of environmental factors (biotic and abiotic) on habitat selection of marine mesoconsumers is necessary to elucidate the potential impacts of these mid-trophic level organisms, the functioning of marine ecosystems, and their management.

As a remote and minimally impacted system, Shark Bay, Western Australia, provides an excellent setting for the examination of factors influencing the habitat selection of mesoconsumers [35]. Shark Bay's sandflats include three microhabitats that are likely to differ in resource abundance, temperature, and accessibility by an intact population of large predators. In addition, mesoconsumers (rays) are abundant and show clear differences in seasonal microhabitat use patterns [36]. The goal of this study was to investigate mesoconsumer microhabitat selection in relation to environmental factors (both biotic and abiotic) to determine the dynamics of habitat selection by individual species as well as community structure in this system. We examined ray abundance relative to prey abundance, predation risk and predictions based on behavioral thermoregulatory theory, specifically, that rays would select microhabitats with the warmest waters to possibly increase digestive rates or that rays would forage in warm microhabitats and rest in cool microhabitats to maximize energetic gains.

## Methods

### Ethics statement

This research was conducted under authorization by the Florida International University Institutional Animal Care and Use Committee (Animal Welfare Assurance Number A3096-01), Fisheries Western Australia permit 4/05 and Department of Environment and Conservation permits CE002111 and SF006493 and comply with the laws of the United States of America and Australia.

### Study site

**Figure 1:**
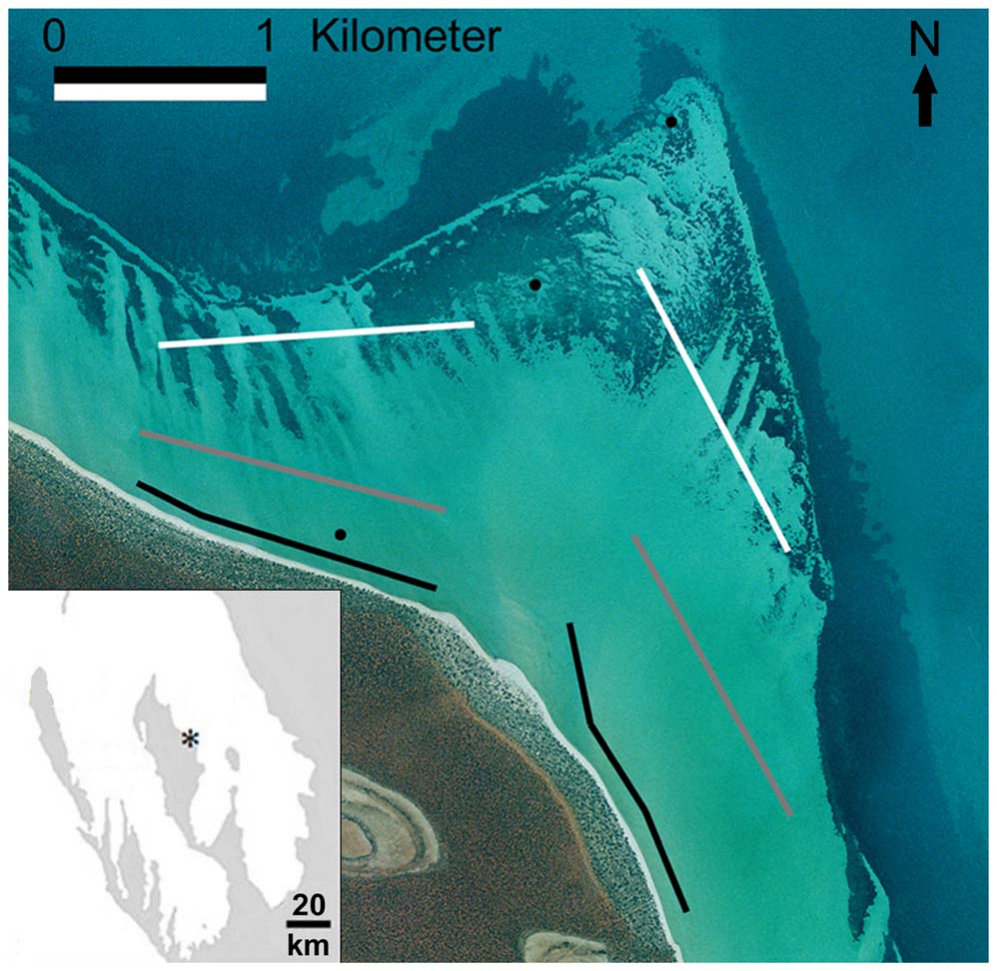
Study site: Cape Rose Flats, Shark Bay, Western Australia. The inset shows the location of the Cape Rose Flats within Shark Bay. The study site was divided into six transects representing nearshore (black), sand (grey), and patchy (white) microhabitats. Black circles represent the location of temperature data loggers.

Shark Bay (25°45′S, 113°44′E) is a large (ca. 13,000 km^2^) semi-enclosed bay on the central west coast of Australia. In addition to vast seagrass shoals, Shark Bay contains several shallow expansive nearshore sandflats with fringing seagrass beds. Within the sandflat habitat of the Cape Rose Flats, three microhabitats have been defined (Fig. 1). With increasing distance from shore, we have designated the microhabitats as nearshore, sand, and patchy. Briefly, nearshore microhabitats are adjacent to the shoreline and intertidal, sand microhabitats have depths of 1–2 m, and patchy microhabitats are 1–3 m deep and are covered with patchy seagrass. A more detailed description of the study site can be found in Vaudo and Heithaus [36]. Throughout the year, juvenile rays of several species make extensive use of these sandflats, particularly the nearshore microhabitat during Shark Bay's warm season (September to May) when sea surface temperatures are greater than 20°C [36].

In this study, we focused on habitat selection by the giant shovelnose ray (*Glaucostegus typus*), http://www.fishbase.org/Nomenclature/SynonymSummary.php?ID=159584&GSID=26787&Status=accepted%20name&Synonymy=senior%20synonym&Combination=new%20combination&GenusName=Glaucostegus&SpeciesName=typus&SpecCode=12577&SynonymsRef=47737&Author=%28Anon%20the reticulate whipray (*Himantura uarnak*) and the pink whipray (*Himantura fai*) because they were the most common rays on the sandflats and previous work identified them as playing a large role in elasmobranch community structure [36]. Within Australia, these three species are found in inshore tropical and subtropical waters, often over shallow soft substrates. In addition, Shark Bay represents the southern limit of their range in Western Australia [37].

### Ray abundance and behavior

To assess habitat selection, we established two 1.5-km long belt transects within each microhabitat (Fig. 1). Between March 2006 and October 2007, we conducted transect sampling from a 4.5-m vessel using the methods described in Vaudo and Heithaus [36]. Briefly, sampling occurred between 08:00 and 16:00 when Beaufort wind conditions were two or less and glare and turbidity did not impair sighting conditions. Vessel speeds did not exceed 6 km h^−1^ and each transect was run only once per day. At the beginning and end of each transect we recorded the sea surface temperature and the predicted tidal height and the means of each of these respective values were used for analyses. All rays within 5 m (or 10 m early in the study [36]) of the transect line were identified and recorded. When possible, we also recorded the behaviors of sighted rays prior to any visible disturbance. On the basis of movements and sightings of tagged rays (J.J. Vaudo, unpublished data), it is unlikely that individual rays were resighted on consecutive passes of a given transect. A total of 181 usable transects were conducted (nearshore: 22 cold season, 31 warm season; sand: 31 cold season, 34 warm season; patchy: 32 cold season, 31 warm season). In addition to recording rays while on transect, after October 2006 the species, positions and behaviors of all elasmobranchs sighted on the Cape Rose Flats were recorded (i.e., those encountered while moving between transects).

Because the transect dataset contained a large number of zeros and is therefore highly skewed, we analyzed ray abundance using conditional models. We first modeled data using a logistic regression for presence/absence. Ray abundance from the zero-truncated data set was then log-transformed and analyzed with a generalized linear model to assess factors influencing ray abundances when they were present on a transect. Factors included in both sets of models were microhabitat, tidal height, and temperature. Models were reduced in a stepwise fashion until only significant factors remained. We performed these analyses separately for the giant shovelnose ray http://www.fishbase.org/Nomenclature/SynonymSummary.php?ID=159584&GSID=26787&Status=accepted%20name&Synonymy=senior%20synonym&Combination=new%20combination&GenusName=Glaucostegus&SpeciesName=typus&SpecCode=12577&SynonymsRef=47737&Author=%28Anon%20and the combined group of reticulate whipray and pink whipray; it was not always possible to distinguish between the two whipray species, although the vast majority are reticulate whiprays [36].

### Prey abundance

To examine the potential influence of prey on ray habitat selection, we examined the abundance of potential prey across microhabitats. Giant shovelnose rays and whiprays in Shark Bay have diets dominated by crustaceans and also include polychaetes [38], so we focused on these taxa. We sampled prey during July 2006 and 2007 (cold season) and September 2006 and March 2007 (warm season). We divided each transect (two per microhabitat) into five equal-area zones and then during each sampling period, selected a random location from each zone and collected two sediment cores (0.15 m diameter×0.2 m deep) using a PVC tube with a plug to create suction. Each random location was only sampled once. Within the patchy microhabitat, 70% of cores were sampled from sand substrate and 30% from seagrass substrate. We sieved each sample through 1-mm mesh to collect potential prey and pooled samples from each location. Potential prey were sorted by taxa and biomass was recorded as wet weight. We used biomass as a measure of abundance because potential prey varied greatly in size, therefore biomass would give a better representation of the amount of prey available than numerical abundance. Prey abundance data for each prey taxa were analyzed separately using conditional models. We first modeled prey taxa presence/absence using a logistic regression. Prey taxa biomass from the zero-truncated data set was then log-transformed and analyzed with a generalized linear model to assess factors influencing biomass when they were present from core samples. Factors included in both sets of models were microhabitat and season (warm and cold). Models were reduced in a stepwise fashion until only significant factors remained.

### Thermal heterogeneity

To examine whether variation in water temperature was related to ray microhabitat selection, we evaluated thermal heterogeneity across the Cape Rose Flats using three temperature loggers (HOBO Water Temp Pro v2, Onset Computer Corporation, accuracy: 0.2°C, resolution: 0.02°C) placed across the sandflat to record bottom temperatures (Fig. 1). Water temperatures were logged every 30 min from 23 April 2007 until the end of the study. We analyzed temperature data with a nested ANOVA, using season and location as factors and day as a blocking factor nested within season. Because the temperature data span two different warm seasons, we considered each warm season separately.

### Predator abundance

To examine whether predator abundance was related to ray microhabitat selection, we assessed tiger shark (*Galeocerdo cuvier*) catch rates throughout the study. Shark fishing was part of a long-term shark survey in Shark Bay conducted approximately in an area approximately 6 – 12 km east of the Cape Rose Flats [39], [40] and as such is meant to show shark presence at a system wide level. Although not concurrent with this study, previous fishing efforts adjacent to the Cape Rose Flats resulted in similar catch rates to those in the core fishing area sampled during this study (M.R. Heithaus, unpublished data). Over the course of this study, shark fishing took place 2.82±1.89 d/month (mean±standard deviation) using the methods described in Wirsing et al. [39]. Up to ten drumlines, each with a single 13/0 Mustad Shark Hook baited with Australian salmon (*Arripis truttaceus*), or local fish when Australian salmon was not available, were fished at a depth of 0.7–2.0 m. Lines were spaced 300–400 m apart and were checked approximately every two hours. We calculated soak time as the time between when the hook was set and retrieved. If bait was missing or a shark was caught, we considered bait removal to take place halfway between the previous check and time the missing bait or shark was observed. We analyzed tiger shark catch rates using conditional models. We first modeled data using a logistic regression for presence/absence. Shark catch rate from the zero-truncated data set was then analyzed with a two-sample t-test assuming unequal variances. Season (warm and cold) was the factor included in these models.

All analyses were performed using JMP 8 (SAS Institute, Inc.).

## Results

### Ray abundance and behavior

**Figure 2:**
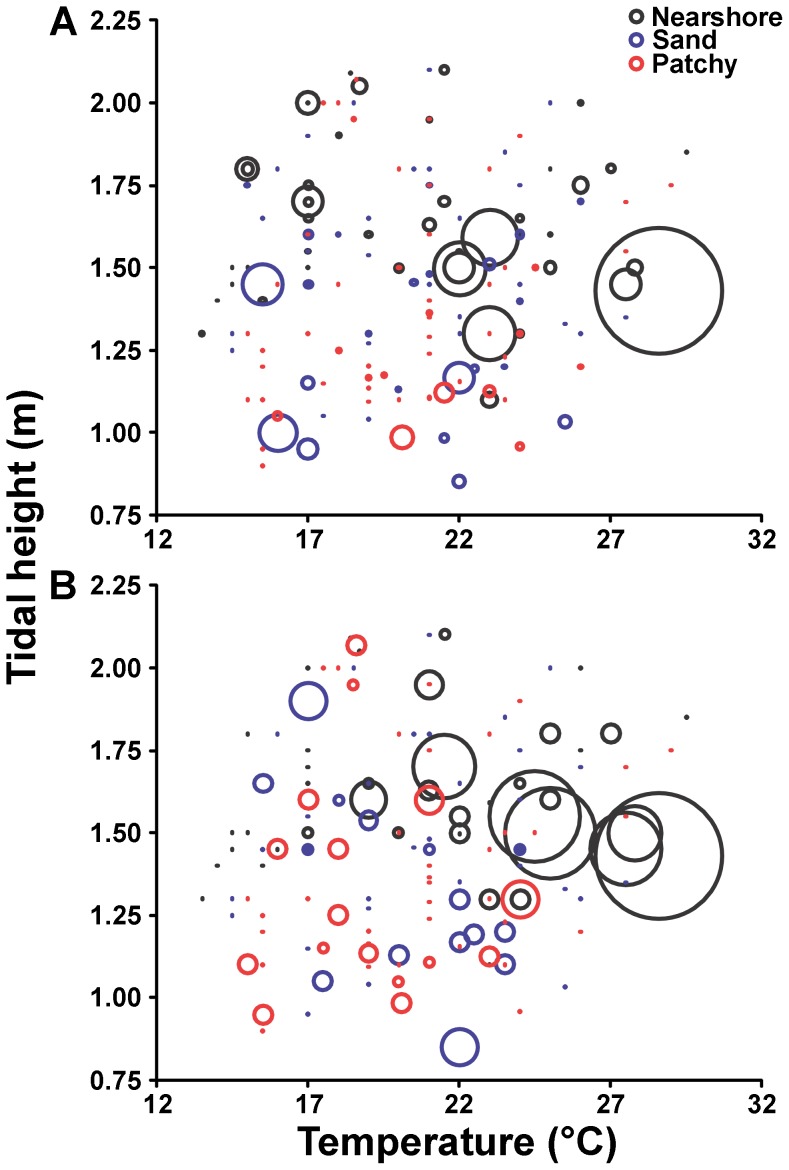
Ray densities. Bubble chart of giant shovelnose ray (*Glaucostegus typus*) (A) and whipray (*Himantura uarnak* and *H. fai*) (B) densities with tidal height, water temperature, and microhabitat. Bubble widths are relative to the maximum density observed for each species group (giant shovelnose ray: 22.67 rays ha^−1^, whipray: 4.67 rays ha^−1^). Dots represent transects in which no rays were observed.

Giant shovelnose ray presence across the sandflat was affected by the interactions of microhabitat×temperature and temperature×tidal height(G = 46.61, d.f. = 7, p<0.001). Giant shovelnose ray occurrence tended to decrease with distance from shore (i.e., nearshore > sand > patchy), and except at temperatures lower than ∼16°C, decreased with tidal height (Table 1). When present, giant shovelnose ray abundance was influenced by a habitat×temperature×tidal height interaction (χ^2^ = 35.81, d.f. = 8, p<0.001). The highest abundances occurred in the nearshore microhabitat and within this microhabitat increased with temperature and decreasing tidal height (Fig. 2A). In sand and patchy microhabitats, densities tended to increase with decreasing tidal height and temperature (Fig. 2A).

**Table 1:** Matrix of predicted probabilities of giant shovelnose ray (*Glaucostegus typus*) occurrence per microhabitat for selected temperatures and tidal heights.

	Temperature (°C)		
	15	20	25
**Tidal Height (m)**	**Nearshore/Sand/Patchy**	**Nearshore/Sand/Patchy**	**Nearshore/Sand/Patchy**
**1**	0.463/0.359/0.122	0.917/0.633/0.430	0.993/0.841/0.803
**1.5**	0.510/0.403/0.144	0.754/0.324/0.173	0.901/0.253/0.207
**2**	0.557/0.449/0.169	0.461/0.117/0.055	0.367/0.021/0.016

Whipray presence was related to microhabitat×temperature and temperature×tidal height interactions (G = 41.02, d.f. = 7, p<0.001). At temperatures below ∼18°C the probability of encountering whiprays on transects increased with tidal height and distance from shore. This pattern reversed during warm periods (Table 2). When whiprays were present, their abundance was influenced by a microhabitat×temperature×tidal height interaction (χ^2^ = 36.41, d.f. = 11, p<0.001). Within the nearshore microhabitat, whipray densities increase with increasing temperature and decreasing tidal height, while in the patchy microhabitat densities increase with temperature and tidal height (Fig. 2B). Whipray densities tend to increase within the sand microhabitat with increasing temperature and decreasing tidal height, but also with increasing tidal height at low temperatures (Fig. 2B).

**Table 2:** Matrix of predicted probabilities of whipray (*Himantura uarnak* and *H. fai*) occurrence per microhabitat for selected temperatures and tidal heights.

	Temperature (°C)		
	15	20	25
Tidal Height (m)	Nearshore/Sand/Patchy	Nearshore/Sand/Patchy	Nearshore/Sand/Patchy
1	0.020/0.128/0.319	0.611/0.387/0.375	0.992/0.732/0.435
1.5	0.070/0.352/0.634	0.408/0.217/0.208	0.862/0.124/0.038
2	0.219/0.668/0.865	0.232/0.108/0.103	0.245/0.007/0.002

Three behaviors (resting, swimming, and foraging) were observed for 750 giant shovelnose rays and whiprays, although only two rays appeared to be foraging when first encountered. Throughout the year, a large majority of giant shovelnose rays and reticulate whiprays across all microhabitats were resting. Pink whiprays, however, were more often observed swimming (Table 3).

**Table 3:** Percentage (sample size) of resting giant shovelnose ray (*Glaucostegus typus*), reticulate whipray (*Himantura uarnak*), and pink whipray (*H. fai*) for each season and microhabitat.

	Nearshore	Sand	Patchy
	Cold/Warm	Cold/Warm	Cold/Warm
Glaucostegus typus	87.0 (54)/82.7 (260)	86.5 (52)/62.9 (62)	69.2 (26)/61.1 (18)
*Himantura fai*	25.0 (4)/38.6 (57)	0.0 (1)/8.3 (72)	−/100.0 (1)
*Himantura uarnak*	87.5 (8)/84.7 (85)	100.0 (7)/77.8 (27)	87.5 (8)/66.7 (6)

### Prey abundance

Most individual prey taxa were rarely encountered, so we pooled taxa into broad taxonomic categories (polychaetes and crustaceans) for statistical analyses. Nine (two polychaete and seven crustacean), 15 (six polychaete and nine crustacean), and 25 (seven polychaete and 18 crustacean) potential prey taxa were recorded from nearshore, sand, and patchy microhabitats, respectively.

Polychaete presence was not influenced by season or microhabitat (G = 8.13, d.f. = 5, p = 0.15). When polychaetes were present, their biomass differed with microhabitat (F = 3.64, d.f. = 2, p = 0.03) with lower biomass found in patchy microhabitats (wet weight: 6.52±0.97/1.15 g m^−2^; mean±lower standard error/upper standard error) than in sand microhabitats (11.44±1.50/1.73 g m^−2^) (Tukey's test t = 2.62, p = 0.03). Although biomass estimates within nearshore microhabitats (10.06±1.33/1.54 g m^−2^) did not differ statistically from the other microhabitats, they were more similar to values from sand microhabitats.

Crustacean occurrence during invertebrate surveys was influenced by season with a higher probability of occurrence during the warm season (warm season: 57%; cold season: 29%) (G = 15.11, d.f. = 5, p = 0.01). When crustaceans were present, their biomass differed between seasons and microhabitats (F = 6.56, d.f. = 1, p = 0.01 and F = 4.08, d.f. = 2, p = 0.02, respectively). Biomass was higher in the cold season (cold season: 3.84±1.62/2.79 g m^−2^; warm season: 0.71±0.22/0.31 g m^−2^) (Tukey's test t = 2.56, p = 0.01) and higher in patchy microhabitats (6.63±3.12/5.88 g m^−2^) than in sand microhabitats (0.61±0.27/0.50 g m^−2^) (Tukey's test t = 2.73, p = 0.02). Nearshore microhabitat values were intermediate (1.11±0.41/0.66 g m^−2^), but more similar to sand microhabitat values.

### Thermal heterogeneity

**Figure 3:**
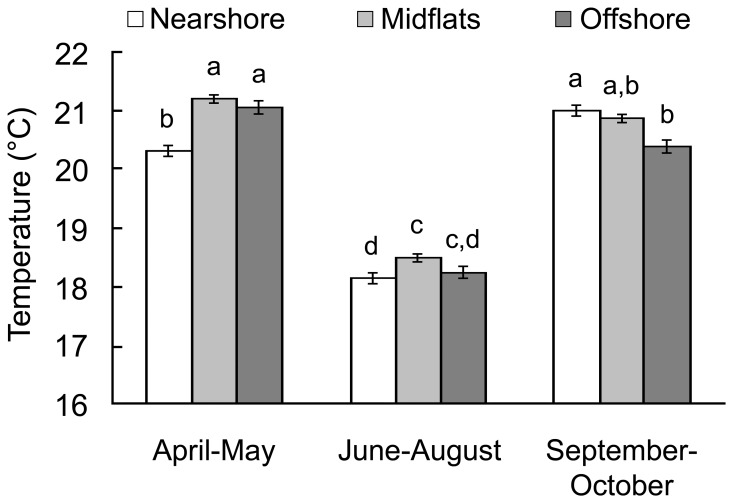
Sandflat temperatures. Seasonal temperatures (mean±standard error) per microhabitat for the time period between 23 April 2007 and 14 October 2007. Bars with different letters are significantly different at *P*<0.01.

**Figure 4:**
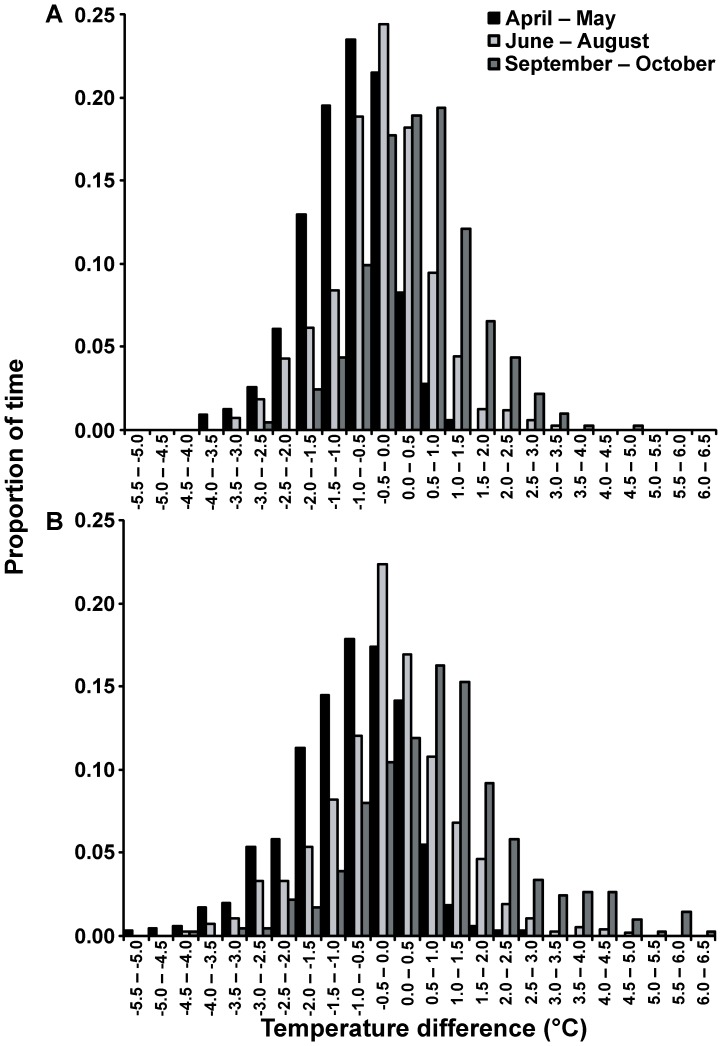
Microhabitat temperature differences. Histogram of temperature differences between nearshore and midflats (A) and nearshore and offshore (B) areas of Cape Rose Flats between 23 April 2007 and 14 October 2007. Negative temperature differences indicate nearshore areas were cooler and positive values indicate nearshore areas were warmer. All recorded temperature values were used for the construction of the histograms.

Between 23 April and 14 October 2007, temperatures on the sandflat ranged from 12.4°C to 27.7°C (nearshore: 12.4°C – 27.7°C; midflats: 14.0°C – 25.7°C; offshore: 15.3°C – 23.4°C), although we recorded temperatures as high as 32.6°C in the nearshore microhabitat during early March 2007. Because of autocorrelation between consecutive temperature readings, statistical analysis of the water temperature data was restricted to the reading taken at 12:00 each day for simplicity; this time was selected because it was the midpoint of the daily sampling period (08:00 – 16:00). Differences in mean temperature were driven by an interaction between season and location (F = 11.39, d.f. = 4, p<0.001). Overall, water temperatures were cooler across the sandflat during the cold season (Tukey's tests, all p<0.001). During the cold season (June – August), temperatures were highest in the interior of the sandflat. Between April and May, temperatures tended to be coolest nearshore, while temperatures were highest nearshore between September and October (Fig. 3). Temperature differences between microhabitats were highly variable over the course of the study, but followed a similar temporal pattern to mean temperature, with the nearshore microhabitat tending to be warmest between September and October (Fig. 4). The temperature gradients (nearshore-offshore) across the sandflat for April – May, June – August, and September – October ranged from −5.1 to 2.6°C, −4.3 to 4.9°C, and −4.5 to 6.2°C, respectively. This difference between warm seasons (April – May and September – October) is likely the result of seasonal changes in air temperature having a greater effect on the temperature of the shallow waters of the nearshore microhabitat. In fact, nearshore temperatures generally exceeded temperatures in the midflat between February and April (J.J. Vaudo, unpublished data).

### Predator abundance

The probability of catching at least one tiger shark per day was higher during the warm season (91%) than during the cold season (41%) (G = 13.46, d.f. = 1, p<0.001). In addition, for days in which sharks were caught, catch rates of tiger sharks were higher in the warm season (0.05±0.01 sharks h^−1^) than cold season (0.03±0.01 sharks h^−1^; t = 2.21, d.f. = 21, p = 0.039).

## Discussion

Many factors contribute to the habitat choice of organisms. Given the variety of biotic and abiotic factors involved, it is likely that these factors will influence habitat choice in different and perhaps even contradictory ways. Understanding how these factors interact to affect habitat choice, however, is necessary to elucidate the role of organisms within systems and how systems may respond to abiotic and biotic changes.

Both giant shovelnose ray and whipray presence and abundance varied with microhabitat, water temperature, and tidal height. At moderate to higher temperatures, typical of the warm season, frequency of occurrence of both groups decreased with distance from shore and also decreased with increasing tidal height. The magnitude of the decrease also increased with temperature such that in the warmest months, rays were rarely found in sand and patchy microhabitats during higher tides. In addition to increases in occurrence, both groups also increase in abundance with increases in temperature in the nearshore microhabitat. The nearshore microhabitat, therefore, appears to be important for these animals when temperatures are high and increases in importance with decreasing tidal height.

The importance of the nearshore microhabitat to Shark Bay's rays appears to have little to do with prey abundance. Higher prey biomass can be found in other sandflat microhabitats. Polychaete abundance does not differ between nearshore and sand microhabitats, and crustaceans, which are the most important component of the diets of giant shovelnose rays and whiprays in Shark Bay [38], [41], had higher biomasses during the cold season and in patchy microhabitats. Previous studies on the invertebrate fauna of Shark Bay's nearshore environment also suggest that potential giant shovelnose ray and whipray prey are more likely to be found in the seagrass beds than on the sandflat [42], [43]. The lack of prey in a microhabitat selected by rays indicates that some other factor besides prey abundance is driving microhabitat choice of the rays, although the possibility that ray foraging has decreased prey abundance warrants investigation.

In many systems, competition drives habitat selection resulting in inferior competitors foraging in less productive habitats [4], [7]. Crustaceans, especially penaeid shrimp, which are important to the diets of giant shovlnose rays and whiprays [38], [41], are also important to the diets of a wide variety of fishes [44], [45] suggesting competition for crustacean prey may occur. If rays are inferior competitors, they may be displaced from the habitats with the highest abundance of prey, creating the mismatch in microhabitat and prey biomass observed. However, despite their high abundance in the nearshore microhabitat, rays were rarely observed foraging during the day and were most often resting. Further, the prey most often encountered in the nearshore microhabitat are rarely found in the stomach contents of giant shovelnose rays and whiprays ([38], J.J. Vaudo, unpublished data), suggesting the nearshore microhabitat is not an important foraging habitat for these species; therefore, selection of the nearshore microhabitat is not likely to be the result of competitive displacement.

The nearshore microhabitat is also the shallowest of the sandflat microhabitats and therefore experiences the greatest temperature fluctuations across the sandflat. These temperature fluctuations may lead to the thermally heterogeneous nature of the sandflat observed during this study. In addition, the sandflat is likely to differ in temperature from the deeper areas of Shark Bay. Given that rays are poikilothermic and that the thermoelectric properties of the gel of the ampullae of Lorenzini may allow elasmobranchs to detect temperature differences of as little as 0.001°C [46], microhabitat choice may be a means of behavioral thermoregulation to exploit thermal gradients.

Like in other poikilotherms, behavioral thermoregulation has been suggested to explain the behaviors of several elasmobranch species. Because elasmobranch development occurs more rapidly at higher temperatures [47], it has been suggested that aggregations of female elasmobranchs in warm waters were capitalizing on increased embryonic development rates to decrease gestation times [48], [49]. This claim has been further bolstered by experimental evidence that pregnant females prefer warmer temperatures [50] and proof that many warm-water aggregations are composed of pregnant females [51]. Similar behaviors have also been suggested in breeding female loggerhead turtles (*Caretta caretta*) within a thermally heterogeneous environment [10]. The sandflat populations of giant shovelnose rays, reticulate and pink whiprays, however, are almost entirely composed of juveniles [36] and cannot benefit in this manner.

Juveniles may be able to benefit by seeking out warmer waters to aid in digestion as has been observed in a variety of teleosts [19], [52]. Higher temperatures lead to increased rates of gastric evacuation in elasmobranchs [53], [54], which are associated with the return of appetite [55]. Shorter gastric evacuations times would allow individuals to resume feeding sooner, allowing for increased intake rates and ultimately may lead to higher growth rates [19]. Therefore, resting in warm waters during digestion would be most beneficial to animals that feed frequently, as opposed to intermittently [56]. Although >60% giant shovelnose and whiprays sampled at the study site contained stomach contents [38], the stomach content volumes for the majority of rays were far less than the rays with the largest stomach content volumes, suggesting the majority of rays were not feeding frequently (J.J. Vaudo, unpublished data) and may not benefit from increased rates of digestion. Further, while temperatures on the sandflats are likely higher than temperatures in deeper waters during the warm season, rays do not appear to seek out the warmest microhabitats on the sandflat, which, given the tropical distributions of these ray species, would not be expected to exceed their optimal temperature ranges. If the thermal heterogeneity patterns observed in 2007 are consistent from year to year, microhabitat choice should differ between the latter portion of the warm season preceding the cold season and the beginning of the following warm season. No such changes are apparent in the data. During the cold season, rays are also least often observed in the warmest sandflat microhabitat.

Energetic gains could also be realized by shuttling between warm and cool waters. By moving into cooler waters, rays could reduce their standard metabolic rate and conserve valuable energetic resources. Resting and energetically expensive processes, such as digestion, should therefore take place in cool waters. Such an energy conservation strategy has been used to explain the movement patterns of bat rays (*Myliobatis californica*) [57] and small-spotted catsharks (*Scyliorhinus canicula*) [20]. Experimental and energetic data also support the use of this tactic in elasmobranchs. When presented with a thermal gradient, Atlantic stingrays (*Dasyatis sabina*) sought out cooler waters after feeding [50] and a temperature increase of 0.9°C was enough to drive small-spotted catsharks away from a food patch between feedings [20]. Further, examinations of Atlantic stingray gut evacuation and absorption rates across a range of temperatures show that decreases in evacuation rate (i.e., food staying in the gut longer) as a result of lower temperatures more than offset concomitant decreases in absorption rates resulting in higher overall absorption [56]. Such a strategy might also be necessary for some active elasmobranchs such as juvenile sandbar shark (*Carcharhinus plumbeus*), for which routine metabolic rate may approach 100% of their metabolic scope [58].

Interestingly, habitat selection by rays in Shark Bay does not follow this pattern. With increasing temperatures, ray abundance increases on the sandflat and the vast majority of giant shovelnose rays and reticulate whiprays on the sandflat are resting, despite cooler waters being available. Even on the finer scale within the sandflats these rays fail to conform to the proposed energy conservation strategy. Giant shovelnose rays and whiprays were most common in the nearshore microhabitat, although it is the warmest microhabitat for at least portions of the warm season.

Thermoregulation via behavioral mechanisms, however, is not without costs [59]. And some costs, such as predation risk, may outweigh the benefits of thermoregulation. Predation risk has been shown to alter the thermoregulatory behaviors of reptiles [60]–[62] and although it is often overlooked as a potential driver of elasmobranch habitat selection, may influence microhabitat choice by giant shovelnose rays and whiprays during portions of the year. The increase in ray abundance in the nearshore microhabitat coincided with an observed increase in catch rates of tiger sharks during this study, which mirrored previously observed seasonal increases of tiger sharks and great hammerhead shark (*Sphyrna mokarran*) in Shark Bay ([39], J.J. Vaudo, personal observation), both of which are ray predators [63], [64]. During the warm season these large sharks are abundant in Shark Bay and can be sighted swimming over the sandflats, although rarely in the nearshore microhabitat [36]. Further suggesting that predation risk plays a role in ray microhabitat selection is the increase in ray densities within the nearshore microhabitat with decreasing tidal height. The dorsoventrally flattened rays can easily move into the very shallow nearshore waters at low tidal heights, but tiger and great hammerhead sharks are restricted by depth, thereby creating a refuge microhabitat. At higher tidal heights, however, ray predators can access nearshore microhabitats and closely approach the shoreline (J.J. Vaudo, personal observation), which may actually constrain escape options for rays. Selection of shallow waters for predator avoidance has been suggested for other elasmobranchs including juvenile lemon shark (*Negaprion brevirostris*) [65], [66] and juvenile blacktip reef shark (*Carcharhinus melanopterus*) [67]. The presence of anti-predator grouping behaviors in Shark Bay by cowtail stingray (*Pastinachus atrus*) [68], [69], which are similar in size to reticulate and pink whiprays, further suggests that selection of the nearshore microhabitat may be an anti-predator behavior.

Although such anti-predator behaviors may result in fewer individuals being eaten, the consequences of anti-predator behaviors can lead to reductions in population size [70], [71] and may be exacerbated by temperature effects. By using the sandflats during the warm season, the rays may experience higher temperatures than they would select in the absence of predators and for at least portions of the warm season the nearshore waters are the warmest waters available. Although the increased metabolic rate resulting from the higher temperature can result in higher growth rates if coupled with increased energy intake (frequent feeding), the low volumes of prey found in their stomachs suggests rays may not be feeding frequently (see above). As a result, rays selecting the nearshore microhabitat to minimize predation risk will probably realize lower growth rates because of the higher metabolic costs combined infrequent foraging. Decreasing growth rates have been observed in a variety of fish species with increasing temperatures [72], [73] and safer habitat choices [74]. Some individuals may even experience weight loss during the warm season, which may explain occasional sightings of gaunt rays on the sandflats (J.J. Vaudo, personal observation). An analogous situation occurs in the small-spotted catshark. Females form daytime refuging aggregations in warmer shallower waters to avoid harassment from males despite the warmer waters having a negative impact on egg production [75].

Anti-predator behaviors are common in nature and, as mesoconsumers, rays are likely to influence the behaviors of their prey as well. Therefore, the presence of tiger and great hammerhead sharks in Shark Bay may indirectly affect lower trophic levels through the alteration of ray microhabitat selection. Yet, the role of tiger sharks in the Shark Bay ecosystem is not limited to affecting rays and potentially their prey. The presence of tiger sharks influences the behaviors of a variety of taxa [40]. And in the cases of green turtle (*Chelonia mydas*) and dugong (*Dugong dugon*), both large seagrass grazers, have the potential to influence the structure of seagrass beds [76]–[78]. The effect of large sharks on rays reinforces that in this system large sharks appear to be keystone species and that their influence may extend beyond the seagrass beds where they are commonly observed and into the sandflats. Further studies are required to elucidate the potential indirect effects of tiger sharks mediated by changes in the behavior of ray mesoconsumers.

The possibility that large sharks may impact structure of neighboring habitats they do not regularly use by altering the habitat selection of potential prey is of particular concern given worldwide shark declines ([79], but see [80]) and warrants further attention. This study also highlights the potential effect of global climate change on mesoconsumers. Climate change has the potential to alter interaction strengths, thereby affecting trophic cascades [81]. In this system, rays may incur temperature related costs as a result of choosing safe habitats. Increasing temperatures may reduce the benefit of nearshore waters to the point that they are no longer a viable refuge. In addition to higher metabolic costs associated with higher temperatures, rays would experience greater exposure to predators, both of which would negatively affect ray populations and potentially have cascading effects. Refuge loss has been observed in experimental work from a grassland food web that contains two spatially segregated predatory spiders. One of the spider species shifted habitats in response to increased temperatures and became prey of the other spider and ultimately altered the structure of the system [30].

Despite microhabitat selection by giant shovelnose rays and whiprays that roughly mimics reported cases of behavioral thermoregulation, predation risk appears to drive the observed microhabitat choice in Shark Bay. During the warm season, when large sharks are abundant, rays move into the coastal sandflats to rest during the day. In particular, rays, especially at low tidal heights, select the shallow nearshore microhabitats, which is the least accessible to large sharks. Because of the high temperatures experienced in the nearshore microhabitat, the benefits of predation-sensitive microhabitat choice by rays may be diminished because of the higher metabolic costs incurred. Because thermoregulatory and predation-sensitive behaviors can benefit poikilothermic organisms, but depending on the thermal environment may result in similar or different habitat choices, it is necessary that studies of habitat selection of mesoconsumers in thermally heterogeneous environments consider both temperature and predation risk, especially considering ongoing environmental change such as large predator decline and climate change.
